# Comparison of corneal elevation and pachymetry measurements made by two state of the art corneal tomographers with different measurement principles

**DOI:** 10.1371/journal.pone.0223770

**Published:** 2019-10-16

**Authors:** Simon Schröder, Achim Langenbucher, Jens Schrecker

**Affiliations:** 1 Institute of Experimental Ophthalmology, Saarland University, Homburg/Saar, Germany; 2 Department of Ophthalmology, Rudolf-Virchow-Klinikum Glauchau, Glauchau, Germany; Nicolaus Copernicus University, POLAND

## Abstract

**Purpose:**

To compare corneal tomography measurements (elevation and pachymetry) as made by two corneal tomographers: Pentacam AXL and CASIA 2.

**Material and methods:**

The devices were used in a standard measuring mode. 77 normal eyes were measured five times with both devices. The data maps for anterior and posterior corneal elevation and pachymetry were exported and analyzed. Repeatability and average values were calculated for each valid data point on the exported data maps. We also calculated a corrected repeatability of the elevation data maps by removing rotation, tilt, and decentration through realignment of the elevation measurement of each eye prior to analyzing the variations in the measurement usingthe same method as for the repeatability.

**Results:**

Pentacam AXL offered the better (corrected) repeatability for anterior corneal elevation measurements. CASIA 2 offered better repeatability for the pachymetry measurements. The tomographers could not be used interchangeably. The central corneal thickness was measured 9 μm ± 3 μm larger when measured with Pentacam AXL compared to CASIA 2.

## Introduction

The anterior segment of the eye can be examined with the help of corneal topographers and tomographers. These are fundamental diagnostic devices in modern ophthalmology with the main purpose of analyzing the shape of the cornea. Precise measurements are crucial for diagnosis, and for control of corneal pathologies and diseases: Corneal tomographers represent the gold standard in detection and classification of corneal ectatic diseases [1,2]. Screening for corneal ectatic diseases such as keratoconus is an essential part of the preoperative diagnostics before any refractive surgery [3]. Anterior corneal topography is a standard method for contact lens fitting [4].

Precise measurements of corneal shape are highly important for the selection of intraocular lens implants (IOLs) in the context of cataract surgery [5]. The posterior corneal surface should not be neglected especially when selecting toric IOLs for the correction of corneal astigmatism [6]. Numerical ray tracing can be used to select or design IOLs which correct higher order aberrations in individual eyes, provided that accurate and precise corneal tomography measurements are available [7,8]. Devices that combine high resolution corneal measurements with biometry, such as the Pentacam AXL, can be used for IOL calculation via numerical ray tracing.

Variations in corneal elevation measurements might be partially due to uncertainties on the position of the eye during measurement [9,10]. Such misalignment can substantially impair the repeatability of corneal tomography.

The purpose of this paper is to compare repeatability, with and without correction of misalignment and mean values of the elevation and pachymetry data maps obtained by two corneal tomographers for normal eyes.

## Materials and methods

The local ethics committee (Sächsische Landesärztekammer) approved the study (EK-BR-33/18-1) which adhered to the guidelines of the declaration of Helsinki. 77 adult volunteers were included in the study. All subjects were able to fixate on the fixation target of each corneal tomographer. Subjects with a history of corneal refractive surgery, with corneal pathologies (such as corneal ectasia), infectious diseases, and/or problems with dry eyes (sicca symptoms) were excluded. To ensure stable corneal conditions, users of soft contact lenses were required to abstain from contact lens use for at least 2 weeks prior to the measurements. Users of hard contact lenses were excluded.

Two corneal tomographers were compared in this study: a Scheimpflug imaging device (Pentacam AXL, Oculus Optikgeräte GmbH, Wetzlar, Germany) and an anterior segment optical coherence tomography (OCT) device (CASIA 2, Tomey Corporation, Nagoya, Japan). One eye of each patient was measured five consecutive times with each device. The subjects were asked to blink before each measurement and to keep their eyes wide open during measurement while fixating on the fixation target. The device was realigned before each measurement. Each eye was measured with both devices within a single session keeping the time difference between repeated measurements and between measurements made with both devices minimal.

Both corneal tomographers were used with standard settings. To reduce the influence of the operator, the tomographers were used in automatic release mode, which means that the measurement started as soon as positioning requirements for the respective tomographer were within the limits predefined by the manufacturer. The Pentacam AXL acquired 25 Scheimpflug images within each measurement. The Pentacam AXL includes a software whose export function provides elevation and pachymetry data maps in Cartesian coordinates with 0.1 mm sampling-resolution in horizontal and vertical direction and 1 μm in the direction along the keratographic axis. The CASIA 2 measurements were obtained with the Corneal Map mode of the pre-op Cataract module. Measurements were exported as data maps in cylindrical coordinates with an angular separation of 11.25°, and a radial resolution of 0.02 mm. With both devices, only measurements with a quality status QS = ‘OK’ were accepted. Invalid data points were excluded. Only eyes with five successful measurements with both tomographers were considered in the analysis.

**Fig 1:**
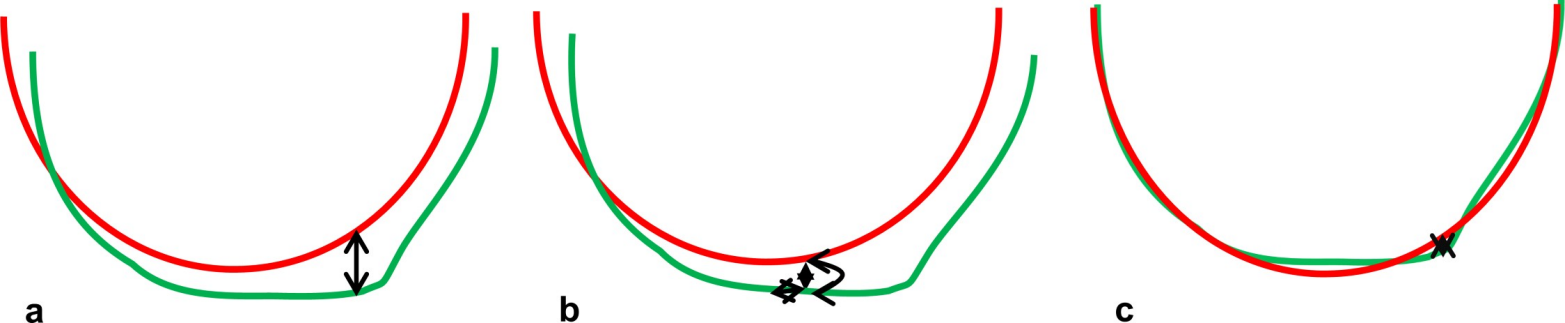
Difference between repeatability and corrected repeatability. Sketch of a central cut through two corneal surface elevation measurements (first measurement in red, second measurement in black) of the same eye. The sketch is not to scale and differences are exaggerated for better visibility. **(a)** Differences in surface elevation between consecutive measurements define the repeatability. **(b)** The differences are partly due to alignment errors such as decentration, axial displacement, rotation, and tilt (misalignment). **(c)** After realignment of the surfaces, the differences are potentially reduced. The differences between the realigned consecutive measurements define the corrected repeatability.

Repeatability refers to the variation between repeated measures of the same eye under the same conditions. We distinguish between repeatability and corrected repeatability: The repeatability is expressed as the within-subject standard deviation (SDw) of the corneal tomography (elevation and pachymetry) measurements without correction of misalignment. The corrected repeatability is the SDw of measurements after correction of misalignment (rotation, translation) between consecutive measurements. (Fig 1 illustrates the impact of misalignment.) Correction of misalignment was performed using the fifth measurement as a landmark to realign the other four measurements. To enable direct comparison between repeatability and corrected repeatability, the fifth measurement was not included in the calculation of repeatability and corrected repeatability.

The repeatability was calculated for each valid data entry on the elevation and pachymetry data maps of Pentacam AXL and CASIA 2 separately. Differences between consecutive measurements were used to calculate the SDw [11] as a measure for repeatability.

For both tomographers, the effect of misalignment was corrected using the elevation of the fifth measurement of each eye as a landmark to realign the other measurements of the same eye. The first four measurements were realigned by applying rigid transformations (rotation and translation) to the elevation data. The rigid transformations minimized the sum of squared differenceswith the fifth measurement for each elevation data-map of the first four measurements separately. To facilitate this process, the fifth measurement was approximated by a weighted sum of the first Zernike polynomials (j<22) [12]. This realignment procedure has been described previously [10]. To calculate the corrected repeatability the realigned elevation data maps had to be interpolated at the original coordinates (natural interpolation using the *scatteredInterpolant* function of MATLAB software with Delaunay Triangulation [13]). This resulted in invalid entries through interpolation of points where no measurement data was available. These invalid entries were removed. The SDw of the realigned measurements was calculated in the same way as for the calculation of the repeatability. The SDw of the of each valid data entry on the realigned elevation data maps measured by Pentacam AXL and CASIA 2 defines the corrected repeatabilitydata entrydata maps.

To analyze systematic differences between the measurements on the two tomographers, the average elevation and pachymetry data maps of each device were used. The differences between the average data maps were calculated considering only the data which both tomographers measured successfully for all measurements of all eyes. To subtract the average data maps of CASIA 2 from the average data maps of the Pentacam AXL, CASIA 2’s measurements were interpolated at the coordinates of the measurement by the Pentacam AXL. All five measurements per eye were considered in the calculation of the average data maps.

## Results

We obtained five valid measurements from 77 eyes of 77 adult volunteers (Table 1) with Pentacam AXL and CASIA 2 –i.e.in total 385 measurements with each tomographer were included in the analysis. On average the volunteers had an age of 73.3 ± 9.1 years (range: 48 to 89 years). Out of the 77 volunteers 41 were women and 36 men. The measurements were used to compare repeatability, and corrected repeatability and to study systematic differences between the measurements made with the two tomographers.

**Table 1:** Corneal characteristics of the study population measured with CASIA 2 expressed as mean, ± standard deviation.

N = 77 eyes (41 right, 36 left eyes)	Kmean	Astigmatism	Thickness
**Anterior Cornea**	48.89 D ±1.62 D, (45.49..51.66) D	0.90 D ± 0.61 D, (0.07..3.21) D	--
**Posterior Cornea**	-6.13 D ± 0.24 D, (-6.63..-5.73) D	0.25 D ± 0.12 D, (0.03..0.57) D	--
**Total Cornea**	42.88 D ± 1.44 D, (39.63..45.39) D	0.82 D ± 0.54 D, (0.08..2.66) D	546.8 μm ±30.6 μm, (470..611) μm

Kmean: simulated average keratometry value, Astigmatism: difference between the simulated keratometry values. The simulated keratometry values were computed based on the radius of curvature at a radial distance of 1.5 mm from the corneal center. The range is given in parentheses.

**Fig 2:**
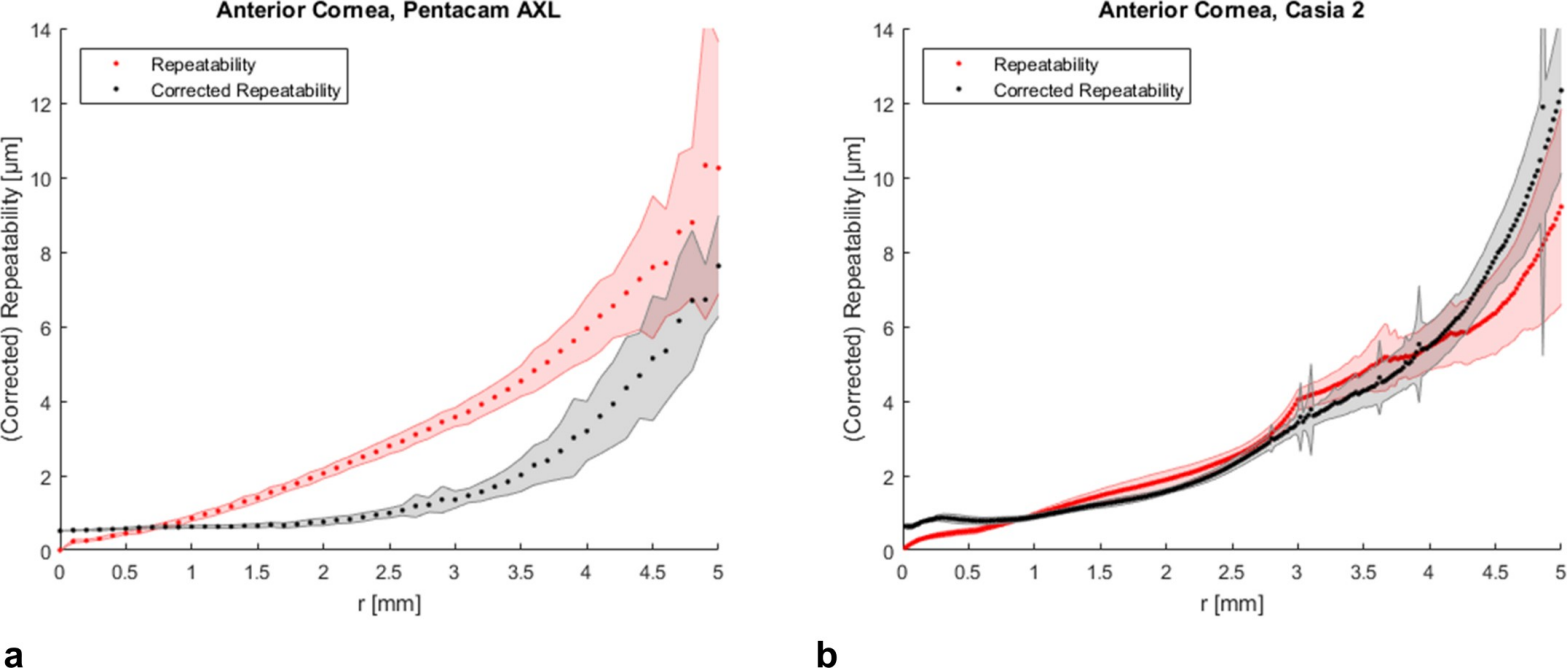
Repeatability and corrected repeatability of anterior corneal elevation. **(a)** Repeatability (red) and corrected repeatability (black) of anterior corneal elevation data maps obtained with Pentacam AXL and **(b)** CASIA 2 averaged along concentric rings ± standard deviation (shaded area) as a function of the radial distance *r* from the corneal apex.

**Fig 3:**
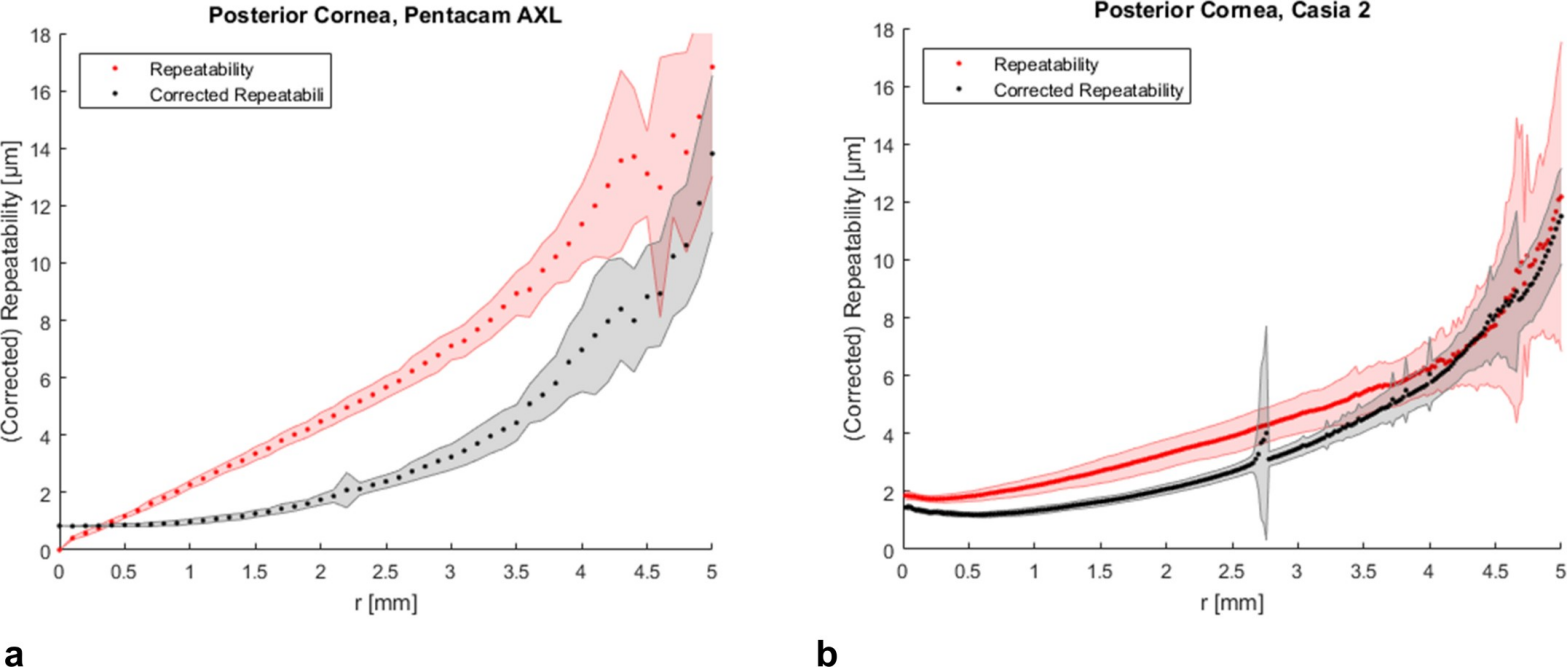
Repeatability and corrected repeatability of posterior corneal elevation. **(a)** Repeatability (red) and corrected repeatability (black) of posterior corneal elevation data maps obtained with Pentacam AXL and **(b)** CASIA 2 (right) averaged along concentric rings ± standard deviation (shaded area) as a function of the radial distance *r* from the corneal apex.

**Fig 4:**
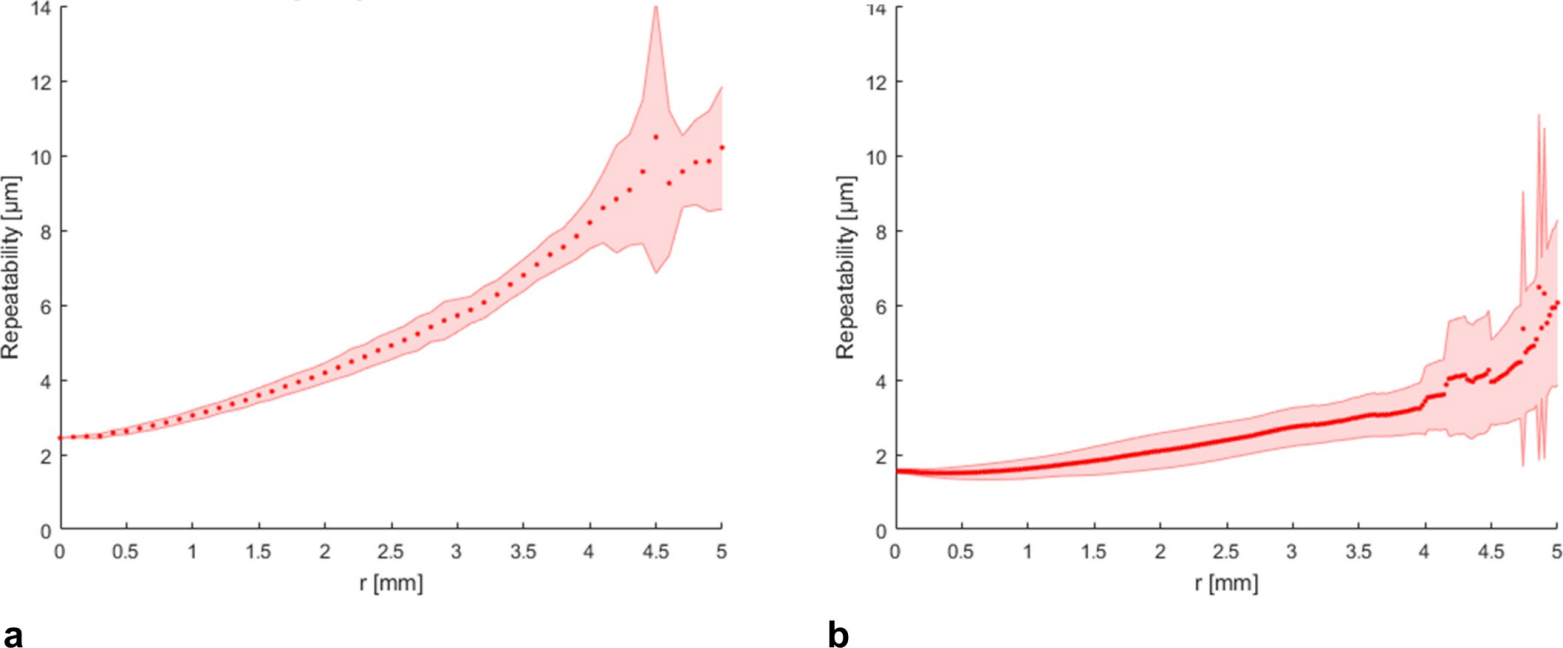
Repeatability of pachymetry. **(a)** Repeatability of pachymetry data maps obtained with Pentacam AXL (left) and **(b)** CASIA 2 (right) averaged alongconcentric rings ± standard deviation (shaded area) as a function of the radial distance *r* from the corneal apex.

The repeatability of all measurements made with both tomographers was best in the center and worst in the periphery (Table 2, Figs 2, 3 and 4). Both tomographers achieved similar repeatability for anterior corneal elevation within the central 8 mm of the cornea (Fig 2). Posterior corneal elevation measurements were less repeatable than anterior corneal elevation measurements and similar between Pentacam AXL and CASIA2 in the central corneal region (Fig 3). The pachymetry measurements with CASIA 2 showed a SDw < 4 μm and were thus very repeatable (Fig 4).

**Table 2:** Repeatability (in μm) averaged along concentric rings for both devices ± standard deviation at 0 mm, 1 mm, 2 mm, 3 mm, and 4 mm distance from the corneal apex for anterior and posterior cornea measured with Pentacam AXL and CASIA 2.

Radial Position	Pentacam AXL			CASIA 2		
	Anterior	Posterior	Pachymetry	Anterior	Posterior	Pachymetry
0 mm	0.0*	0.0*	2.44 ± 0.11	0.0*	1.74 ± 0.11	1.52 ± 0.09
1 mm	0.86 ± 0.08	2.28 ± 0.13	3.05 ± 0.13	0.94 ± 0.07	2.23 ± 0.29	1.62 ± 0.26
2 mm	2.06 ± 0.15	4.46 ± 0.30	4.19 ± 0.27	1.89 ± 0.23	3.30 ± 0.49	2.10 ± 0.47
3 mm	3.56 ± 0.27	7.08 ± 0.50	5.72 ± 0.44	4.03 ± 0.28	4.64 ± 0.63	2.74 ± 0.51
4 mm	5.92 ± 0.80	11.3 ± 1.3	8.20 ± 0.70	5.47 ± 0.65	6.23 ± 0.86	3.43 ± 0.90

* The apex always referenced as 0μm.

The corrected repeatability was calculated from the elevation data maps that were corrected for misalignment. There were larger differences between repeatability and corrected repeatability of corneal elevation measurements with Pentacam AXL compared to CASIA 2 (Figs 2 and 3). For the anterior surface, the corrected repeatability was better with Pentacam AXL (Table 3).

**Table 3:** Corrected repeatability (in μm) averaged along concentric rings for both devices ± standard deviation at 0 mm, 1 mm, 2 mm, 3 mm, and 4 mm distance from the corneal apex for anterior and posterior cornea measured with Pentacam AXL and CASIA 2.

Radial Position	Pentacam AXL		CASIA 2	
	Anterior	Posterior	Anterior	Posterior
0 mm	0.52	0.84	0.64 ± 0.07	1.44 ± 0.07
1 mm	0.63 ± 0.03	0.99 ± 0.09	0.90 ± 0.07	1.33 ± 0.13
2 mm	0.76 ± 0.10	1.75 ± 0.18	1.58 ± 0.08	2.08 ± 0.18
3 mm	1.36 ± 0.23	3.24 ± 0.45	3.42 ± 0.37	3.48 ± 0.28
4 mm	3.19 ± 0.80	6.97 ± 1.46	5.47 ± 0.63	6.06 ± 1.29

**Fig 5:**
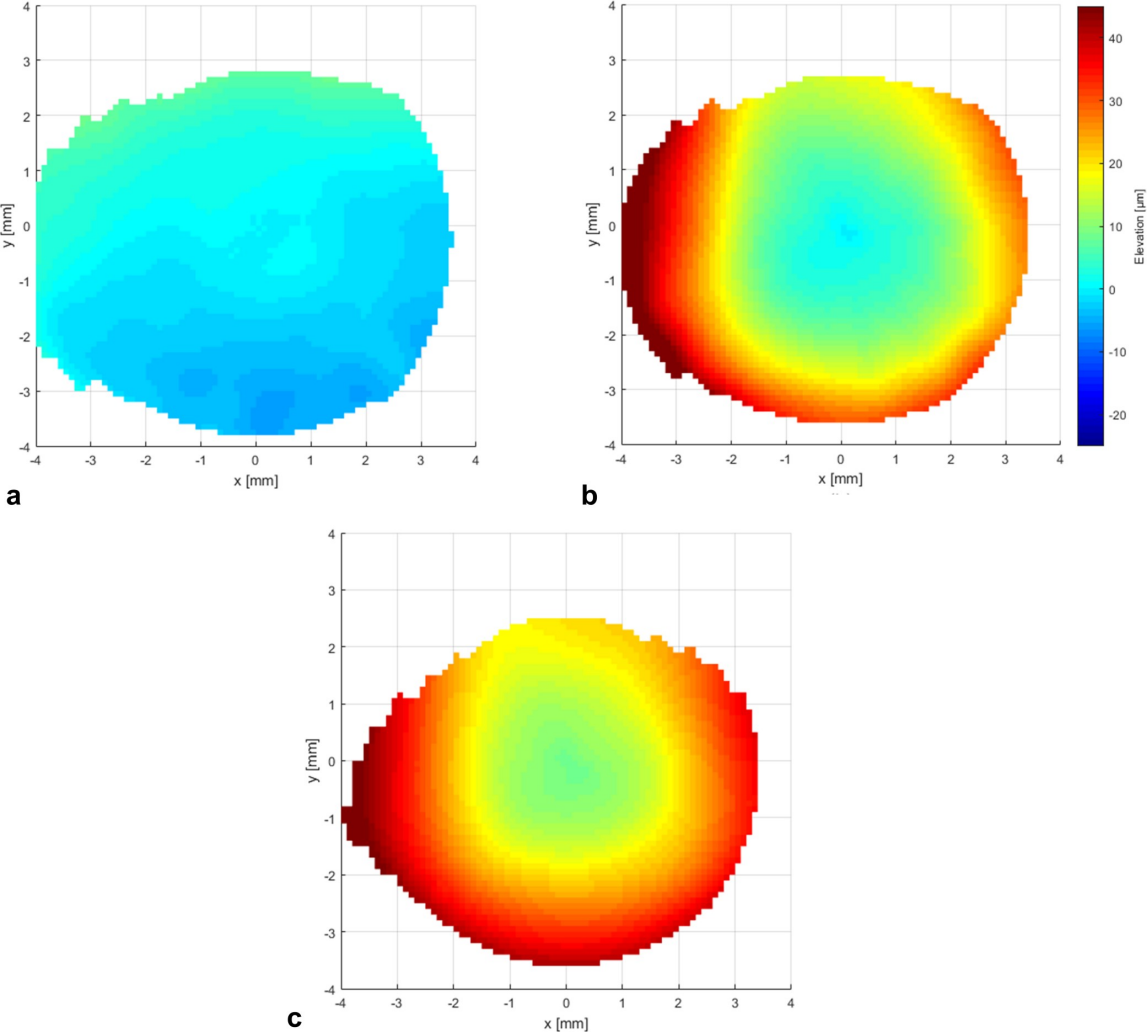
Systematic differences. Systematic differences between the average elevation data maps obtained with Pentacam AXL and CASIA 2 (average elevation obtained with Pentacam AXL minus average elevation with CASIA 2). **(a)** The measurements are consistent for the anterior corneal elevation. **(b)** There were significant systematic differences between the posterior corneal elevation measured with Pentacam AXL and CASIA 2 and **(c)** between the pachymetry measurements.

The average values of the anterior corneal elevation measurements with Pentacam AXL and CASIA 2 were consistent (Fig 5, Table 4). Within the central 8 mm, the average difference between the anterior corneal elevation measurements by Pentacam AXL and CASIA 2 was -0.2 μm ± 3.1 μm (mean ± SD). The respective difference for posterior corneal elevation was 18 μm ± 11 μm after subtracting the central corneal thickness included in the measurements by CASIA 2. The pachymetry measurements showed the largest differences with an average value of 26 μm ± 10 μm. The differenceat the corneal apex was 8.6 μm ± 3 μm. The differences between measurements with both devices were smaller in the center than in the periphery.

**Table 4:** Mean elevation and pachymetry values (in μm) averaged along concentric rings for both devices ± standard deviation at 0 mm, 1 mm, 2 mm, 3 mm, and 4 mm distance from the corneal apex measured with Pentacam AXL and CASIA 2.

	Pentacam AXL			CASIA 2		
r	Anterior	Posterior**	Pachymetry	Anterior	Posterior	Pachymetry
**0 mm**	0*	0.41	555.36	0.02 ± 0.01	546.96 ± 0.73	546.93 ± 0.61
**1 mm**	65.61 ± 0.70	80.00 ± 6.24	563.33 ± 5.26	64.76 ± 0.34	622.11 ± 5.23	551.31 ± 4.72
**2 mm**	264.81 ± 2.12	324.55 ± 13.43	588.57 ± 10.13	264.75 ± 0.53	859.52 ± 12.34	568.69 ± 10.59
**3 mm**	607.56 ± 3.85	742.57 ± 14.92	625.38 ± 10.39	606.81 ± 1.40	1267.12 ± 16.65	595.72 ± 14.52
**4 mm**	1106.9 ± 3.0	1362.3 ± 3.6	674.48 ± 2.75	1106.6 ± 3.5	1864.5 ± 5.5	627.84 ± 8.3

* Apex referenced with 0.0 μm

**) Posterior elevation with Pentacam AXL did not include the central corneal thickness.

## Discussion and conclusions

We studied repeatability, corrected repeatability and systematic differences of the elevation and pachymetry data maps obtained with Pentacam AXL and CASIA 2 for 77 normal eyes from 77 subjects. Pentacam AXL had better repeatability for anterior corneal elevation, whereas CASIA 2 had much better repeatability for the pachymetry. Misalignment limited the repeatability of the elevation data maps of Pentacam AXL. The devices could not be used interchangeably because of significant systematic differences between measurements.

CASIA 2 and Pentacam AXL are relatively new instruments. The Pentacam AXL is similar to the Pentacam HR, which uses the same measurement principle for corneal tomography, but does not offer axial length measurement. There are several studies discussing repeatability of measurements with Pentacam HR. Mac Alinden et al. [14] obtained repeatability of pachymetry and corneal elevation 2, 3, and 4 mm inferior to the corneal apex. They reported repeatability values that were slightly larger than the values obtained in our study, which may be related to repeatability being better in the horizontal than in the vertical direction. For the pachymetry in the central region, repeatability between 3.55 μm and 4.47 μm were reported previously [10,14–16], which is worse than our estimate (2.4 μm). Pentacam AXL measured the pachymetry with a repeatability similar to that of the posterior corneal elevation. In a previous study we looked at repeatability, corrected repeatability and systematic differences between Pentacam HR and CASIA SS-1000 [10]. Pentacam AXL and CASIA 2 each had a performance very similar to their respective predecessor device (Pentacam HR or CASIA SS-1000).

The Pentacam AXL showed superior repeatability and corrected repeatability values for the anterior corneal surface compared to CASIA 2. In the center of the cornea, this is probably in part because of the way its internal algorithm maps the measurements onto a regular grid in Cartesian coordinates for the export of the elevation data maps. In doing so, the resolution in the center is reduced. This might improve the repeatability if multiple measurement points were combined to one value on the elevation data-map.

With both devices, the anterior cornea was measured with lower statistical uncertainty (repeatability and corrected repeatability) than the posterior cornea. However, due to the smaller difference in refractive indices at the posterior corneal surface boundary compared to the anterior corneal surface, the statistical uncertainty of the anterior surface has a larger impact on the optical properties of the cornea and is responsible for >75% of the statistical uncertainty in the tomography based corneal wavefront estimation.

CASIA 2 provided excellent repeatability for the pachymetry, which was much smaller than the repeatability for posterior corneal surface measurements. This result is consistent with the repeatability of central corneal thickness measurements with CASIA SS-1000 [10,17].

Misalignment was found to be an important limitation for the repeatability of the measurements with Pentacam AXL. The amount of misalignment (decentration, axial displacement, tilt, rotation) was of the same magnitude as observed in a previous study with Pentacam HR [10], and with a similar model of the tomographer [9]. The misalignment of the anterior and posterior corneal surfaces was treated independently, because it was not known how each device corrects the optical distortions of the posterior surface when measuring through the anterior cornea. The misalignment of the posterior surface tended to be larger. If we had used combined correction of anterior and posterior corneal misalignment, the differences between repeatability and precision would have been reduced. The corrected repeatability calculated after misalignment correction was comparable with the values of Pentacam HR and CASIA SS-1000 obtained previously [10].

Our study provides a direct comparison between the two measurement devices. The exported data maps (elevation or pachymetry) from the Pentacam AXL often covered a larger region (≥ 42.8 mm^2^) of the cornea than the measurements from CASIA 2 (≥ 24.7 mm^2^). The analysis of systematic differences considered only the region that could be measured with both devices for all measurements of all eyes. This region of approximately 6 mm in diameter is larger than typical photopic pupil diameters [18]. The anterior corneal elevation maps of both tomographers were very similar, but the differences in posterior corneal elevation and pachymetry measurements were larger and exceeded the measurement repeatability. Pentacam AXL reported thicker pachymetry measurements than CASIA 2. The difference of approximately 9 μm in the central pachymetry measurements by the two tomographers is consistent with the differences between measurements of central corneal thickness with Pentacam HR and CASIA SS-1000 [10,19].

The differences between the measurements with Pentacam AXL and CASIA 2 increase towards the corneal periphery. This results in differences of corneal curvature, especially for the posterior cornea. However, the optical differences in the measurements of the anterior corneal radius have a larger impact on the optical properties because of the larger difference in the refractive indices at the anterior corneal boundary compared to the posterior corneal boundary [20]. Based on a conicoidal surface fit to the central 6 mm of the average elevation, we estimated that the difference in refractive power between the measurements with Pentacam AXL is about -0.21 D for the anterior cornea and about -0.14 D for the posterior cornea. data mapsConsistent with the simulated keratometry differences of ~0.1 D observed between Pentacam HR and CASIA SS-1000 for keratoconic eyes [19] we observed that measurements by Pentacam AXL provided smaller refractive corneal power values compared to the measurements by CASIA 2.

The elevation differences between consecutive measurements with the same device are expected to be partially caused by variations of the tear film thickness [21]. Fluctuations of corneal shape can exceed the repeatability of the corneal measurements on a long timescale but within the timescale of our measurements these are expected to have negligible influence on our assessment of repeatability and corrected repeatability. [22,23].

In this study we included only eyes without corneal pathologies. It is very likely that the measurements would be less repeatable and show worse (corrected) repeatability for patients with corneal shape abnormalities such as keratoconus [24]. Systematic differences between the elevation and pachymetry measurements with Pentacam AXL and CASIA 2 might also be greater for pathological corneas.

In conclusion, Pentacam AXL and CASIA 2, showed comparable repeatability and corrected repeatability with Pentacam HR and CASIA SS-1000, respectively. The Pentacam AXL offered superior (corrected) repeatability for the anterior cornea elevation data maps compared to CASIA 2. CASIA 2 offered good (corrected) repeatability for posterior corneal elevation data maps, and superior repeatability for the pachymetry measurements. The devices cannot be used interchangeably due to systematic differences. When pachymetry is critical, doctors and clinical staff should be aware of the systematic differences between the devices.

## Supporting information

S1 File The file contains two Excel spread-sheets. The spreadsheets CASIA2.xlsx and Pentacam AXL.xlsx contain the results obtained with CASIA 2 and Pentacam AXL respectively.
(RAR)

## References

[1] Lopes BT, Ramos IC, Dawson DG, Belin MW, Ambrósio R. Detection of ectatic corneal diseases based on pentacam. Z Med Phys. 2016; 26: 136–142. 10.1016/j.zemedi.2015.11.001 26777318

[2] Prakash G, Agarwal A, Mazhari AI, Kumar G, Desai P, Kumar DA, et al A new, pachymetry-based approach for diagnostic cutoffs for normal, suspect and keratoconic cornea. Eye. 2012; 26: 650–657. 10.1038/eye.2011.365 22281864 PMC3351046

[3] Wilson SE, Klyce SD. Screening for Corneal Topographic Abnormalities before Refractive Surgery. Ophthalmology. 1994; 101: 147–152. 10.1016/s0161-6420(94)31372-8 8302548

[4] Szczotka LB, Roberts C, Herderick EE, Mahmoud A. Quantitative descriptors of corneal topography that influence soft toric contact lens fitting. Cornea. 2002; 21: 249–255. 10.1097/00003226-200204000-00003 11917171

[5] Norrby S. Sources of error in intraocular lens power calculation. J Cataract Refract Surg. 2008; 34: 368–376. 10.1016/j.jcrs.2007.10.031 18299059

[6] Preussner P-R, Hoffmann P, Wahl J. Impact of Posterior Corneal Surface on Toric Intraocular Lens (IOL) Calculation. Curr Eye Res. 2015; 40: 809–814. 10.3109/02713683.2014.959708 25259550

[7] Schrecker J, Langenbucher A, Seitz B, Eppig T. First results with a new intraocular lens design for the individual correction of spherical aberration. J Cataract Refract Surg. 2018; 44: 1211–1219. 10.1016/j.jcrs.2018.06.055 30120004

[8] Packer M, Fine IH, Hoffman RS. Aspheric intraocular lens selection based on corneal wavefront. J Refract Surg. 2009; 25: 12–20. 10.3928/1081597X-20090101-03 19244948

[9] Bao F, Wang J, Huang J, Yu Y, Deng M, Li L, et al Effect of Misalignment between Successive Corneal Videokeratography Maps on the Repeatability of Topography Data. PLoS One. 2015; 10: e0139541 10.1371/journal.pone.0139541 26599442 PMC4658180

[10] Schröder S, Mäurer S, Eppig T, Seitzs B, Rubly K, Langenbucher A. comparison of corneal tomography. Repeatability, precision, misalignment, mean elevation, and mean pachymetry. Curr Eye Res. 2018; 43: 709–716. 10.1080/02713683.2018.1441873 29482368

[11] Synek V. Evaluation of the standard deviation from duplicate results. Accred Qual Assur. 2008; 13: 335–337. 10.1007/s00769-008-0390-x

[12] Noll RJ. Zernike polynomials and atmospheric turbulence. J Opt Soc Am. 1976; 66: 207.

[13] Amidor I. Scattered data interpolation methods for electronic imaging systems: a survey. Journal of Electronic Imaging. 2002; 11: 157–176.

[14] McAlinden C, Khadka J, Pesudovs K. A comprehensive evaluation of the precision (repeatability and reproducibility) of the Oculus Pentacam HR. Invest Ophthalmol Vis Sci. 2011; 52: 7731–7737. 10.1167/iovs.10-7093 21810981

[15] Piotrowiak I, Soldanska B, Burduk M, Kaluzny BJ, Kaluzny J. Measuring Corneal Thickness with SOCT, the Scheimpflug System, and Ultrasound Pachymetry. ISRN Ophthalmol. 2012; 2012: 869319 10.5402/2012/869319 24558594 PMC3914255

[16] Chen S, Huang J, Wen D, Chen W, Huang D, Wang Q. Measurement of central corneal thickness by high-resolution Scheimpflug imaging, Fourier-domain optical coherence tomography and ultrasound pachymetry. Acta Ophthalmol. 2012; 90: 449–455. 10.1111/j.1755-3768.2010.01947.x 20560892

[17] Neri A, Malori M, Scaroni P, Leaci R, Delfini E, Macaluso C. Corneal thickness mapping by 3D swept-source anterior segment optical coherence tomography. Acta Ophthalmol. 2012; 90: e452–7. 10.1111/j.1755-3768.2012.02453.x 22682316

[18] Schröder S, Chashchina E, Janunts E, Cayless A, Langenbucher A. Reproducibility and normal values of static pupil diameters. Eur J Ophthalmol. 2017: [Epub ahead of print]. 10.5301/ejo.5001027 28885673

[19] Szalai E, Berta A, Hassan Z, Módis L. Reliability and repeatability of swept-source Fourier-domain optical coherence tomography and Scheimpflug imaging in keratoconus. J Cataract Refract Surg. 2012; 38: 485–494. 10.1016/j.jcrs.2011.10.027 22261325

[20] Liou HW B. Anatomically accurate, finite model eye for optical modeling. J Opt Soc Am A. 1997; 14: 1684–1695. 10.1364/josaa.14.001684 9248060

[21] Aranha Dos Santos V, Schmetterer L, Gröschl M, Garhofer G, Schmidl D, Kucera M, et al In vivo tear film thickness measurement and tear film dynamics visualization using spectral domain optical coherence tomography. Opt Express. 2015; 23: 21043–21063. 10.1364/OE.23.021043 26367956

[22] Norrby S, Hirnschall N, Nishi Y, Findl O. Fluctuations in corneal curvature limit predictability of intraocular lens power calculations. J Cataract Refract Surg. 2013; 39: 174–179. 10.1016/j.jcrs.2012.09.014 23158678

[23] Kiely PM, Carney LG, Smith G. Diurnal variations of corneal topography and thickness. Am J Optom Physiol Opt. 1982; 59: 976–982. 10.1097/00006324-198212000-00007 6891565

[24] Zheng Y, Huang L, Zhao Y, Wang J, Zheng X, Huang W, et al Repeatability of corneal elevation maps in keratoconus patients using the tomography matching method. Sci Rep. 2017; 7: 17457 10.1038/s41598-017-17658-7 29234085 PMC5727056

